# Adiposity, fat-free mass and incident heart failure in 500 000 individuals

**DOI:** 10.1136/openhrt-2024-002711

**Published:** 2024-07-04

**Authors:** Ayodipupo S Oguntade, Hannah Taylor, Ben Lacey, Sarah Lewington

**Affiliations:** 1 Clinical Trial Service Unit & Epidemiological Studies Unit (CTSU), Nuffield Department of Population Health, University of Oxford, Oxford, UK; 2 Department of Cardiology, Hull University Teaching Hospitals NHS Trust, Hull, UK; 3 UK Biobank, Stockport, UK; 4 MRC Population Health Research Unit, Nuffield Department of Population Health (NDPH), University of Oxford, Oxford, UK

**Keywords:** HEART FAILURE, Obesity, Epidemiology

## Abstract

**Background and aims:**

The independent role of body fat distribution and fat-free mass in heart failure (HF) risk is unclear. We investigated the role of different body composition compartments in risk of HF.

**Methods:**

Present analyses include 428 087 participants (mean age 55.9 years, 44% male) from the UK Biobank. Associations of long-term average levels of body composition measures with incident HF were determined using adjusted Cox proportional hazards regression models.

**Results:**

Over a median follow-up of 13.8 years, there were 10 455 first-ever incident HF events. Overall, HF risk was more strongly associated with central adiposity (waist circumference (WC) adjusted for body mass index (BMI); HR 1.38, 95% CI 1.32 to 1.45) than general adiposity (BMI adjusted for WC; HR 1.22, 95% CI 1.16 to 1.27). Although dual X-ray absorptiometry-derived body fat remained positively related to HF after adjustment for fat-free mass (HR 1.37, 95% CI 1.18 to 1.59), the association of fat-free mass with HF was substantially attenuated by fat mass (HR 1.12, 95% CI 1.01 to 1.26) while visceral fat (VAT) remained associated with HF independent of subcutaneous fat (HR 1.20, 95% CI 1.09 to 1.33). In analyses of HF subtypes, HF with preserved ejection fraction was independently associated with all fat measures (eg, VAT: HR 1.23, 95% CI 1.12 to 1.35; body fat: HR 1.36, 95% CI 1.17 to 1.57) while HF with reduced ejection fraction was not independently associated with fat measures (eg, VAT: HR 1.29, 95% CI 0.98 to 1.68; body fat: HR 1.29, 95% CI 0.80 to 2.07).

**Conclusions:**

This large-scale study shows that excess adiposity and fat mass are associated with higher HF risk while the association of fat-free mass with HF could be explained largely by its correlation with fat mass. The study also describes the independent relevance of body fat distribution to HF subtypes, suggesting different mechanisms may be driving their aetiopathogenesis.

**WHAT IS ALREADY KNOWN ON THIS TOPIC**

- Obesity as defined by the body mass index is a risk factor for heart failure (HF) but the independent role of body fat distribution and fat-free mass using whole body imaging in incident HF and its subtypes risk is unclear.

**WHAT THIS STUDY ADDS**

- Central adiposity especially visceral fat is an independent risk factor for HF while the association of fat-free mass with HF is explained by its correlation with fat mass. HF with preserved ejection fraction was independently associated with excess visceral and body fat while HF with reduced ejection fraction was not.

**HOW THIS STUDY MIGHT AFFECT RESEARCH, PRACTICE OR POLICY**

- These findings may be important for preventative population health strategies for tackling the current obesity pandemic in the general population.

## Introduction

The associations of adiposity, body fat distribution and fat-free mass (collectively termed ‘body composition’) with cardiac dysfunction have been an area of active investigation. The most widely studied measure of body composition is body mass index (BMI), used as a measure of general adiposity.^1–3^ However, BMI is an imprecise measure as it does not differentiate between body fat (and its distribution in different body compartments) and fat-free mass. Moreover, there is emerging evidence that measures of central adiposity may be more strongly associated with heart failure (HF) risk.^4–6^ Furthermore, recent studies have shown that fat-free mass may be more strongly associated with cardiovascular risk than with adiposity measures, but the independent effects of fat-free mass and the various adiposity measures on HF risk are not well established.^7–9^

Anthropometric measures do not directly measure fat mass and fat-free mass and the use of other fat quantification measures like bioelectrical impedance analysis (BIA) and dual X-ray absprtiometry (DXA) could potentially provide insights into the role of fat distribution and fat-free mass in individuals who are at increased risk of HF in the population. To the best of our knowledge, no recent study has examined the relevance of fat and fat-free mass quantification using both BIA and DXA in HF risk, and fat and fat-free mass measurement are not yet routinely used in cardiovascular risk prediction in the general population.

HF can be subtyped using left ventricular ejection fraction (LVEF) obtained from cardiac imaging into HF with preserved ejection fraction (HFpEF), HF with reduced ejection fraction (HFrEF), and, more recently, HF with mildly reduced ejection fraction.^10 11^ Obesity has been shown to be a risk factor for HFpEF in some observational studies but data on HFrEF is sparse.^12–16^ However, LVEF is not commonly recorded in routine data and as such, there is little evidence on the relationship between measures of body composition and HF subtypes in large-scale observational studies. UK Biobank (UKB) and its extensive record linkage provide a good opportunity to investigate the relevance of excess adiposity and fat-free mass in relation to HF and its different subtypes. A better understanding of the aetiology of HF and its subtypes will inform prevention at individual and population levels.

In the present study, we determined the associations between measures of body composition and risk of incident HF and its subtypes. We also explored the independent and joint effects of general and central adiposity on HF. Finally, we explored the independent associations of longitudinal changes in body composition measures with incident HF in this contemporary population cohort.

## Methods

This study followed the Strengthening the Reporting of Observational Studies in Epidemiology reporting guideline.^17^ The design and data collection procedures of UKB have been previously described.^18^ Briefly, the UKB is a prospective cohort of 502 000 adults (aged 40–70 years) recruited from the general population of the UK between 2006 and 2010.^19^ All UKB participants have given written informed consent for the use of their data for health research. A repeat survey of the full baseline assessment including anthropometry and bioimpedance was conducted on approximately 20 000 participants in 2012–2013 and used to calculate regression dilution ratios (RDRs) of these measures as explained below in the statistical analysis section. The UKB imaging substudy started in 2014 and is ongoing.^20^ So far, 57 670 participants without interval HF event between the initial and imaging visits over a mean interval of 10 years have additionally had further anthropometry and bioimpedance remeasured. Alongside a repeat of the baseline assessment, participants of the imaging substudy underwent whole body imaging for the assessment of cardiac function and body composition, using cardiac MRI (CMR) and DXA, respectively. The present analysis included 428 087 individuals with complete anthropometric and bioimpedance measures who were free of HF and other major cardiovascular conditions at the baseline assessment visit. Of these, about 36 000 individuals from the initial baseline assessment have had whole body DXA imaging and 2913 of these individuals have had repeat DXA body imaging.

All UKB participants have their data linked to their UK National Health Service (NHS) records and are followed up by electronic health records linkage (hospital episode statistics and Office for National Statistics cause of death).^21^ All the authors had full access to all the data in the study and took responsibility for its integrity and the data analysis.

### Data collection procedures

At recruitment participants reported lifestyle exposures, medical history and medications before undergoing standardised assessments including body size and biomarker measurements. Anthropometric measurements included body weight (using a Tanita BC418MA body composition analyser), standing height (Seca 240 cm height measure), and waist and hip circumference (Seca 200 cm tape measure around the narrowest part of the trunk and the widest part of the hips, respectively).^19 22 23^ Waist-to-hip ratio (WHR) is the ratio of the waist circumference (WC) to hip circumference (both in cm). BMI is the ratio of the weight (kg) to the square of the height in metres. BIA measures of body fat mass (BFM) and fat-free mass (BFFM) were obtained from the same Tanita BC-418MA analyser. This involved the participants standing barefooted on the analyser’s foot pads while holding onto metal conductor hand grips.^22 23^

During the imaging substudy, DXA (GE-Lunar, Madison, Wisconsin, USA) was used to estimate body fat-free mass and body fat distribution using a proprietor device algorithm.^20^ Briefly, the android region is defined by transverse planes at the top of the iliac crest and at 20% of the distance between the iliac crest and the top of the trunk.^19^ The Gynoid area is the area around the hips defined by transverse planes at 1.5 and 2.5 times the height of the android region below the iliac crest.^19^ Subcutaneous adipose tissue (SAT) was defined as subcutaneous fat in the android and gynoid regions. The amount of SAT in the android region was estimated by measuring the fat between the abdominal wall and the skin line on both sides of the image, and this estimate of subcutaneous abdominal fat was subtracted from the android fat to obtain the visceral fat (VAT) estimate.^24^ DXA-derived fat-free mass was calculated by subtracting the DXA-derived total body fat from total body mass.

### HF incidence in UK Biobank

The primary outcome in this study was a first-ever recorded incident HF diagnosis after the baseline visit (for anthropometric and bioimpedance measures) or first imaging visit (for DXA-derived measures). Incident HF events were coded using the International Classification of Diseases, 10th Revision (ICD-10) and the OPCS Classification of Interventions and Procedures version 4 (OPCS-4). ICD-10 codes used to define HF include I11.0, I13.0, I13.2 and I50. OPCS-4 codes used to define HF procedures are K01 (transplantation of heart and lung), K02 (other transplantation of heart), K54 (Open heart assist operations), K56 (Transluminal heart assist operations) and K76 (Transluminal operations on cardiac conduit). A detailed list of ICD-10 and OPCS-4 codes used in the present analysis can be found in online supplemental table S1.

10.1136/openhrt-2024-002711.supp1 Supplementary data

Participants’ primary care records were used to obtain the HF LVEF phenotype for a fraction of the participants with incident HF (1464 individuals) whose primary care records are linked to the UKB study and who have had echocardiography done in primary care using read codes and SNOMED CT codes (online supplemental table S2). HFrEF was identified from diagnostic codes of systolic dysfunction or reduced LVEF. HFpEF was identified using diagnostic codes of diastolic dysfunction, normal LVEF or preserved LVEF in the absence of concomitant codes for reduced ejection fraction. HF with midrange ejection fraction was not considered because this phenotype was not routinely coded in primary care. This was supplemented by LVEF obtained from CMR in the ongoing UKB imaging substudy where this was available. Thus, there were 901 HFrEF events and 563 HFpEF events which were categorised.

### Statistical analysis

We excluded UKB participants who had withdrawn from the study (n=111), those outside the age group of 40–69 years (n=2431) and those with missing (n=11 894) or outlying body composition measures outside the 0.01th percentile and 99.99th percentiles of the distribution (n=530). Finally, to minimise reverse causation, we excluded individuals with HF at baseline (n=2501) or with other major cardiac and vascular conditions (n=59 747) (see online supplemental figure S1) leaving 428 087 participants in the main analysis. For the analysis of DXA-derived measures, we excluded individuals with interval HF or other major cardiac and vascular conditions at their first imaging visit and included 36 278 individuals in the analyses.

We summarised baseline characteristics of participants in proportions or means (SD). The inter-relationships between body composition measures were determined using Pearson’s partial correlation method (online supplemental tables S3, S4). Participants were followed via electronic health record linkage from the date of recruitment at baseline (or first imaging visit in analyses of DXA measures) until the date of (1) first HF event, (2) death or (3) loss to follow-up. Cox proportional hazard regression models were used to estimate adjusted HRs stratified by age at risk, sex and UK region (England, Wales and Scotland), and further adjusted for ethnicity (European, South-Asian, African and others), education, quintiles of the Townsend deprivation index, smoking (never, past and current smokers), alcohol (lifetime abstainer, ex-drinker, occasional drinker (up to three times per month)) and regular drinker (weekly/daily) and physical activity (<10, 10–49.9, ≥50 metabolic equivalent [MET]-hour/week). For each of these potential confounders, individuals with missing values were assigned to the largest category. Analyses that use values of body composition measures obtained on a single occasion at baseline which do not take into account within person variability over time, are prone to systematic underestimation of the strength of associations between measured body composition and HF risk (‘regression dilution bias’).^25 26^ As such, in this analysis, RDRs were calculated using the age-adjusted and sex-adjusted Pearson partial correlations (r) between baseline and resurvey measures (4.3 years after baseline visit) in 17 450 individuals of the present analysis who attended both visits (online supplemental table S5). In the case of DXA-derived measures, RDRs were calculated using the age and sex adjusted Pearson partial correlations (r) between initial imaging and imaging resurvey measures (2 years after the initial imaging visit) in 2913 individuals who attended both visits (online supplemental table S5). This method has been described in detail elsewhere.^27 28^ Associations were corrected for regression dilution bias by dividing the beta coefficients (and standard errors) by the RDR and described as association with usual (long-term average) body composition measures. Previous population studies have shown the SD of BMI in the general population to be approximately 5 kg/m^2^ and large population studies often express the excess risk attributable to BMI per 5 kg/m^2^ higher.^29–32^ As such in the present analyses, HRs were reported as per usual 5 BMI units equivalent higher of each body composition measure based on the measured SD of the different body composition measures in the UKB as shown in online supplemental table S6 (eg, 5 BMI units is equivalent to 12.2 WC units, 0.07 WHR units, 9.8 BFM units, 4.9 BFFM units, 0.6 VAT units and 2.1 SAT units) so that the strength of associations can be easily compared.

The shapes of the association between ‘usual’ levels of body composition and HF risk were described by plotting the floating absolute risks against the mean body composition at resurvey within each baseline quintiles of each body composition measure.^33^ The variance of the logHR (95% CI) in each group, including the reference, was calculated (from the variances and covariances of the logHR in all groups except the reference group) and used to obtain group-specific logHR (95% CIs) as previously described.^27 33^ HRs (ie, floated absolute risks) were estimated for each quintile with the bottom fifth designated as the reference.

Independent effects of general (BMI) and central adiposity (WC and WHR), and of fat and fat-free mass were investigated by comparing the strength of associations before and after mutual adjustment for each other. In the mutual adjustment analyses, because of the correlations between the body composition measures, the residuals obtained from a first-step linear regression of the body composition measures against each other (which are uncorrelated) were used in the second stage Cox regression analyses. The χ^2^ values of the different models were compared and used to explain which of the different body composition measures was most informative of HF risk. The joint effects of both general and central fat distribution on incident HF were assessed by determining the associations of tertiles of central adiposity measures (WC, WHR and VAT) with incident HF among normal weight, overweight and obese individuals (as defined by BMI). Effect modifications by age at risk (5 years age groups), sex, ethnicity, smoking and alcohol were assessed by including an interaction term in the multivariable model. Heterogeneity between subgroups was assessed using χ^2^ tests for heterogeneity while χ^2^ tests for trend were used to assess if the HRs differed in any ordered subgroups. A semiquantitative estimate of mediation was evaluated for each body composition measure by assessing the change in the χ^2^ statistic for that body composition measure following sequential adjustment for intermediate factors considered to lie on the causal pathway of associations between body composition and HF. Intermediate factors included systolic blood pressure (SBP), type 2 diabetes mellitus (T2DM), blood glucose, glycated haemoglobin (HbA1c), C reactive protein, estimated glomerular filtration rate (eGFR), serum high density lipoprotein cholesterol (HDL-C) and low density lipoprotein cholesterol (LDL-C).

We then determined the associations of decadal longitudinal changes in anthropometric and bioimpedance measures and incident HF from initial visit to imaging visit (mean interval of 10 years) in analyses of subset of participants with repeat body composition measures (57 670 participants) without interval HF event before the imaging visit. Briefly, per cent changes in each body composition measure were calculated ((imaging value–baseline value)/baseline value×100%).^9 34^ Participants were divided into five groups based on percent change in each body composition measure (⊿% <– 8%, –8% ≤⊿% < –2%, –2% ≤⊿% < 2% (reference group), ≥2% ⊿ %<10% and ⊿% ≥10%). Stable body composition was defined as – 2% ≤ ⊿ body composition measure <2% based on previous literature on age-related changes in body fat and fat-free mass.^34–36^

Sensitivity analyses were performed to assess the potential for reverse causality and residual confounding. Separate analyses excluded the first 2 years of follow-up and the shape and strength of associations were compared with those of the main analyses. Analyses excluding underweight individuals were also performed to investigate residual confounding from undernutrition at baseline.

Proportional hazards assumption was tested by checking the Schoenfeld residuals of each body composition measure in the association between HF incidence and natural log of the follow-up time. Considerations were given to multiple testing using a Bonferroni-corrected p value threshold that accounted for the total number of body composition measures tested (ie, p<0.006). All analyses were done using Stata V.17.0 (StataCorp) while plots were made using the Jasper package (developed by Matt Arnold) in R V.4.1.3 (R Foundation for Statistical Computing, Vienna, Austria).

## Results

### Overall incident HF

Over a median follow-up of 13.8 years, there were 10 455 incident HF events in the main analysis of anthropometric and BIA measures while in online supplemental figure S5 analysis of DXA-derived measures, there were 292 incident HF events over a median follow-up of 4 years among individuals without pre-existing HF or major cardiovascular conditions. Participants had a mean (SD) age of 55.9 (8.1) years, 188 312 (44%) were male and 405 177 (94.2%) were European. Overall, there were more HF events in men (6197 vs 4258 incident HF) than women (table 1). On average, those who developed HF were older, more socioeconomically deprived, more likely to be current smokers and had higher adiposity, VAT and fat-free mass. Participants who developed HF were twice as likely to have hypertension and three times more likely to have T2DM than those who did not develop HF and also had higher SBP, diastolic BP, plasma glucose and HbA1c. Participants with HF were also more likely to have chronic kidney disease, be on BP and lipid-lowering medications and insulin than those without HF. Participants with HF also had lower eGFR than participants without HF.

**Table 1:** Participants’ characteristics by incident heart failure in UK Biobank*

Characteristics at baseline	Incident heart failure		
	No	Yes	Total
Baseline visit	N=417 632	N=10 455	N=428 087
Age (years)	55.8 (8.0)	61.4 (6.4)	55.9 (8.1)
Men	182 116 (43.6%)	6196 (59.3%)	188 312 (44.0%)
England	370 390 (88.7%)	9845 (94.2%)	380 235 (88.8%)
European	395 222 (94.6%)	9955 (95.2%)	405 177 (94.6%)
Higher education	269 783 (64.6%)	7079 (67.7%)	276 862 (64.7%)
Most deprived	82 826 (19.9%)	2684 (25.7%)	85 510 (20.0%)
Current smoker	42 349 (10.1%)	1670 (16.0%)	44 019 (10.3%)
Regular/daily drinker	293 908 (70.4%)	6874 (65.7%)	300 782 (70.3%)
Low physical activity	72 643 (17.4%)	1973 (18.9%)	74 616 (17.4%)
BMI (kg/m^2^)	27.2 (4.6)	29.4 (5.6)	27.2 (4.7)
Waist circumference (cm)	89.4 (13.0)	97.4 (14.5)	89.6 (13.1)
Waist-to-hip ratio	0.9 (0.1)	0.9 (0.1)	0.9 (0.1)
BIA-body fat mass (kg)	24.5 (9.3)	27.7 (11.3)	24.5 (9.4)
BIA-body fat-free mass (kg)	52.8 (11.4)	56.8 (12.0)	52.9 (11.4)
Hypertension	99 287 (23.8%)	4880 (46.7%)	104 167 (24.3%)
Diabetes	17 162 (4.1%)	1344 (12.9%)	18 506 (4.3%)
Chronic kidney disease	2298 (0.6%)	161 (1.5%)	2459 (0.6%)
BP lowering medication usage	67 234 (16.1%)	3818 (36.5%)	71 052 (16.6%)
Insulin usage	3190 (0.8%)	341 (3.3%)	3531 (0.8%)
Lipid-lowering medication usage	49 506 (11.9%)	2674 (25.6%)	52 180 (12.2%)
Systolic BP (mm Hg)	137.5 (18.5)	146.2 (19.4)	137.7 (18.6)
Diastolic BP (mm Hg)	82.4 (10.1)	84.3 (10.5)	82.4 (10.1)
Glucose (mmol/L)	5.1 (1.1)	5.5 (1.9)	5.1 (1.2)
HbA1c (mmol/mol)	35.7 (6.1)	38.7 (10.0)	35.8 (6.3)
HDL-C (mmol/L)	1.5 (0.4)	1.4 (0.4)	1.5 (0.4)
LDL-C (mmol/L)	3.6 (0.8)	3.5 (0.9)	3.6 (0.8)
eGFR (mL/min)	90.5 (15.3)	79.9 (17.7)	90.3 (15.4)
C reactive protein (mg/L)†	2.8 (0.7)	2.7 (0.8)	2.8 (0.7)†
DXA imaging visit‡			
DXA-body fat mass (kg)	25.5 (9.3)	27.9 (10.7)	25.5 (9.3)
DXA-visceral fat (kg)	1.2 (0.9)	1.7 (1.2)	1.2 (0.9)
DXA-subcutaneous adipose tissue (kg)	5.3 (2.0)	5.3 (2.1)	5.3 (2.0)
DXA-fat-free mass (kg)	49.7 (10.1)	53.8 (10.7)	49.8 (10.1)

*Data are presented as arithmetic mean (SD), geometric mean (SD) or n (%).

†Geometric mean (SD).

‡Imaging visit occurred 10 years after the baseline visit.

BIA, bioelectrical impedance analysis; BMI, body mass index; BP, blood pressure; DXA, dual X-ray absprtiometry; eGFR, estimated glomerular filtration rate; HbA1c, glycated haemoglobin; HDL-C, High density lipoprotein cholesterol; LDL-C, low density lipoprotein cholesterol.

**Figure 1:**
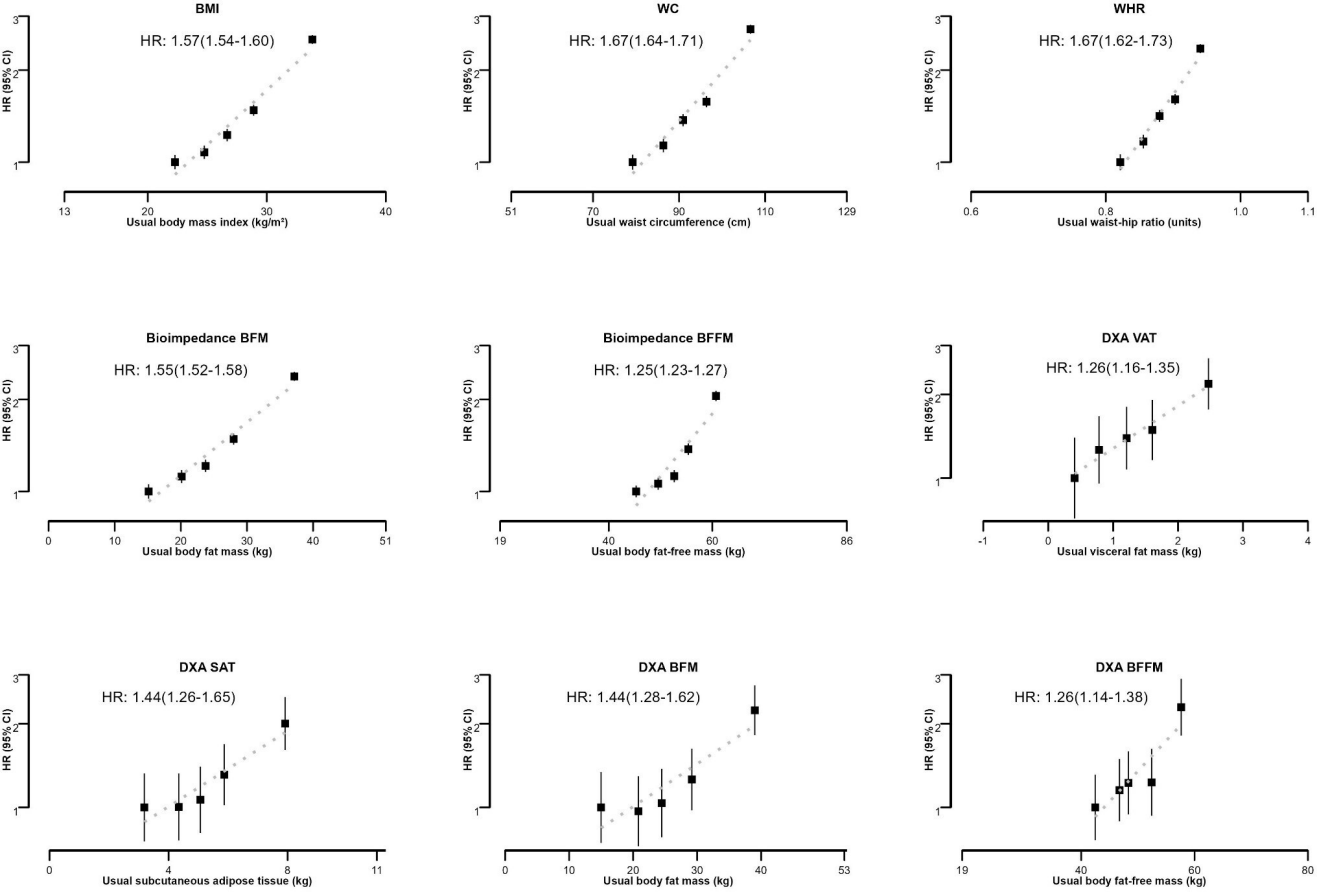
Shape of associations of body composition measures with incident HF in the UK Biobank. Error bars denote group-specific 95% CIs. Group-specific HRs were plotted against the resurvey means of each body composition measure in each baseline quintile. HR estimates were stratified by age at risk (5-year age groups), sex and UK region and were adjusted for ethnicity, education, social deprivation, smoking, alcohol and physical activity. BFM, body fat mass; BFFM, body fat-free mass; BMI, body mass index; DXA, dual X-ray absorptiometry; SAT, subcutaneous adipose tissue; VAT, visceral fat; WC, waist circumference; WHR, waist-to-hip ratio.

All the body composition measures showed positive and log-linear associations with incident HF in both sexes (figure 1). Associations were stronger for usual WC and WHR than BMI or fat measures (table 2). Each 5 kg/m^2^ higher usual BMI was associated with 57% higher HF risk (95% CI 1.54 to 1.60). Both usual WC and WHR were associated with 67% higher HF risk per 12.2 cm (95% CI 1.64 to 1.71) and per 0.07units (95% CI 1.62 to 1.73), respectively. The strength of associations for both usual BIA-derived and DXA-derived body fat was comparable (HR 1.55 and HR 1.44, respectively) and similar to that for BMI. BIA-derived and DXA-derived fat-free mass showed comparable but weaker association with incident HF (HR 1.25 (95% CI 1.23 to 1.27) and HR 1.26 (95% CI 1.14 to 1.38)). Both VAT and SAT were associated with a higher risk of incident HF (HR 1.26 (95% CI 1.16 to 1.35) and HR 1.44 (95% CI 1.26 to 1.65), respectively). Associations did not significantly differ by sex, age, ethnicity, smoking or alcohol consumption (online supplemental figures S2, S3) and were not substantially different from the main analyses when expressed per usual SD of each body composition measure (online supplemental figures S4, S5).

**Table 2:** Independent associations of usual levels of body composition measures with incident heart failure

Models*	HR (95% CI)	P value	χ^2^
Anthropometry			
BMI	1.57 (1.54 to 1.60)	<0.001	1994
+WC	1.22 (1.16 to 1.27)	<0.001	77
WC	1.67 (1.64 to 1.71)	<0.001	1992
+BMI	1.38 (1.32 to 1.45)	<0.001	169
WHR	1.67 (1.62 to 1.73)	<0.001	1083
+BMI	1.30 (1.25 to 1.34)	<0.001	203
Bioimpedance			
Body fat mass	1.55 (1.52 to 1.58)	<0.001	1700
+Fat free mass	1.46 (1.41 to 1.50)	<0.001	617
Fat-free mass	1.25 (1.23 to 1.27)	<0.001	874
+Body fat mass	1.06 (1.04 to 1.08)	<0.001	31
DXA-imaging			
Body fat mass	1.44 (1.28 to 1.62)	<0.001	36
+Fat-free mass	1.37 (1.18 to 1.59)	<0.001	18
Fat-free mass	1.26 (1.14 to 1.38)	<0.001	22
+Body fat mass	1.12 (1.01 to 1.26)	0.04	4
Visceral fat	1.26 (1.16 to 1.35)	<0.001	33
+Subcutaneous adipose tissue	1.20 (1.09 to 1.33)	<0.001	12
Subcutaneous adipose tissue	1.44 (1.26 to 1.65)	<0.001	28
+Visceral fat	1.22 (1.02 to 1.47)	0.03	5

*All models were stratified by age at risk (in 5-year ranges), sex and UK region and were adjusted for ethnicity, education, social deprivation, smoking, alcohol and physical activity.

BMI, body mass index; WC, waist circumference; WHR, waist-hip ratio.

The association of BMI with HF was substantially attenuated when adjusted for WC (HR 1.22 (95% CI 1.16 to 1.27; χ^2^=77)). By contrast, adjustment of WC for BMI attenuated its association with incident HF risk to HR 1.38 (95% CI 1.32 to 1.45; χ^2^=169) while adjustment of WHR for BMI attenuated its association with incident HF risk to HR 1.30 (95% CI 1.25 to 1.34;χ^2^=203) (see table 2).

Adjustment of BIA-derived body fat for fat-free mass did not substantially attenuate its association with incident HF risk (HR 1.46 (95% CI 1.41 to 1.50; χ^2^=617)). However, adjustment of BIA-derived fat-free mass for body fat substantially attenuated its association with incident HF (HR 1.06 (95% CI 1.04 to 1.08; χ^2^=31)) (table 2). Associations of DXA-derived body fat and fat-free mass when mutually adjusted for each other were comparable with the results of the main analyses of BIA-derived measures. Both VAT and SAT remained independently associated with HF risk after mutual adjustment for each other (HR 1.20 (95% CI 1.09 to 1.33; p<0.001)) and (HR 1.22 (95% CI 1.02 to 1.47; p=0.03)), respectively, although the CI for SAT was wider.

Table 3 shows the joint associations of general and central fat distribution with incident HF risk. Across all tertiles of WC, WHR and VAT, there was a graded log-linear higher risk of HF from normal weight to overweight and obese individuals which was higher in individuals in upper tertiles than lower tertiles of each central fat distribution measure.

**Table 3:** Joint associations of general and central fat distribution on heart failure (HF) risk

Body composition	Normal weight		Overweight		Obese	
	HF events	HR (95% CI)	HF events	HR (95% CI)	HF events	HR (95% CI)
Waist circumference						
Tertile 1	1515	1.00 (0.95 to 1.05)	626	1.11 (1.02 to 1.20)	11	1.67 (0.92 to 3.01)
Tertile 2	592	1.21 (1.12 to 1.32)	2143	1.32 (1.26 to 1.38)	278	1.68 (1.49 to 1.89)
Tertile 3	38	1.08 (0.79 to 1.49)	1470	1.63 (1.55 to 1.71)	3742	2.64 (2.56 to 2.73)
Waist-to-hip ratio						
Tertile 1	989	1.00 (0.94 to 1.06)	768	1.20 (1.12 to 1.29)	217	2.25 (1.97 to 2.57)
Tertile 2	777	1.28 (1.20 to 1.38)	1545	1.44 (1.37 to 1.52)	802	2.22 (2.07 to 2.38)
Tertile 3	379	1.40 (1.27 to 1.55)	1926	1.73 (1.65 to 1.81)	3012	3.03 (2.92 to 3.14)
Visceral fat						
Tertile 1	43	1.00 (0.74 to 1.35)	17	1.22 (0.75 to 1.96)	4	3.04 (1.14 to 8.13)
Tertile 2	17	0.84 (0.52 to 1.36)	43	1.07 (0.79 to 1.45)	13	2.37 (1.37 to 4.08)
Tertile 3	3	1.16 (0.37 to 3.61)	45	1.31 (0.98 to 1.75)	70	2.09 (1.65 to 2.65)

HR estimates are floated absolute risks of heart failure and models were stratified by age at risk (5-year age groups), sex and UK region and adjusted for ethnicity, education, social deprivation, smoking, alcohol and physical activity.

All the body composition measures remained positively associated with HF risk in models adjusted for cardiometabolic intermediate factors except for SAT which association was completely attenuated after adjustment for CRP (table 4). SBP, glycaemic status, eGFR and CRP mediated approximately 80% of the association as shown by the percentage reduction in the χ^2^ value. Serum lipids did not appear to be important mediators of the observed associations. The shape and strength of associations between body composition measures and HF did not also differ from the main analyses when the first 2 years of follow-up or underweight individuals were excluded (online supplemental figures S5, S6). Associations did not differ appreciably for fatal HF events and use of BP lowering or lipid-lowering medications had no appreciable effect on the overall estimates (online supplemental figure S7).

**Table 4:** HR (95% CI) for HF per five usual BMI units equivalent higher usual body composition measures with adjustment for potential intermediate risk factors

Models	Model 1		Model 2		Model 3		Model 4	
	HR (95% CI)	χ^2^	HR (95% CI)	χ^2^	HR (95% CI)	χ^2^	HR (95% CI)	χ^2^
Usual levels of anthropometry								
Body mass index	1.57 (1.54 to 1.60)	1994	1.48 (1.45 to 1.52)	1017	1.37 (1.33 to 1.40)	599	1.11 (1.02 to 1.20)	399
Waist circumference	1.67 (1.64 to 1.71)	1992	1.57 (1.52 to 1.61)	1014	1.43 (1.39 to 1.48)	620	1.36 (1.33 to 1.41)	426
Waist-to-hip ratio	1.67 (1.62 to 1.73)	1083	1.49 (1.43 to 1.54)	451	1.38 (1.33 to 1.44)	299	1.32 (1.27 to 1.37)	207
Usual levels of bioimpedance measures								
Body fat mass	1.55 (1.52 to 1.58)	1700	1.46 (1.42 to 1.49)	872	1.34 (1.30 to 1.37)	488	1.27 (1.24 to 1.31)	304
Body fat-free mass	1.25 (1.23 to 1.27)	874	1.19 (1.17 to 1.21)	413	1.15 (1.13 to 1.17)	251	1.13 (1.11 to 1.15)	209
Usual levels of DXA imaging measures								
Visceral fat	1.25 (1.16 to 1.35)	33	1.18 (1.08 to 1.30)	13	1.15 (1.05 to 1.26)	8	1.12 (1.02 to 1.23)	5
Subcutaneous fat	1.44 (1.26 to 1.65)	28	1.29 (1.10 to 1.51)	10	1.22 (1.04 to 1.43)	6	1.15 (0.98 to 1.36)	3
Body fat mass	1.44 (1.28 to 1.62)	36	1.30 (1.13 to 1.50)	13	1.23 (1.06 to 1.43)	8	1.17 (1.00 to 1.36)	4
Body fat-free mass	1.26 (1.14 to 1.38)	22	1.19 (1.07 to 1.33)	10	1.17 (1.05 to 1.30)	8	1.15 (1.03 to 1.28)	7

Model 1: stratified by age at risk (5-year age groups), sex and UK region and adjusted for ethnicity, education, social deprivation, smoking, alcohol and physical activity.

Model 2: model 1+systolic blood pressure, glycated haemoglobin, blood glucose, diabetes, low density lipoprotein cholesterol and high density lipoprotein cholesterol.

Model 3: model 2+estimated glomerular filtration rate.

Model 4: model 3+C reactive protein.

BMI, body mass index; DXA, dual X-ray absprtiometry; HbA1c, glycated haemoglobin; HF, heart failure.

Table 5 shows the associations of decadal change in body composition measures with incident HF. Individuals with ≥10% decadal increase in BMI from baseline had about twofold higher risk of HF compared with individuals with stable BMI. Individuals with more than 8% decadal decrease in BMI from baseline also had a 35% higher risk of incident HF than those with stable BMI while individuals with decadal BMI decrease of 2%–8% or decadal increase of 2%–10% had no significant increased risk of HF when compared with individuals with stable BMI. Decadal changes in each of WC and WHR were not associated with incident HF risk. There was a 33% higher risk of HF among individuals with 2%–8% decadal decrease in body fat but further decrease in body fat or increase in body fat was not associated with higher risk of HF when compared with individuals with stable body fat mass. On the contrary, compared with those with stable fat-free mass, there was a significant graded increase in HF risk with decadal increase in fat-free mass (42% higher HF risk for decadal increase of 2% to <10% and 4-fold higher HF risk for ≥10% decadal increase in fat-free mass). Decadal decrease in fat-free mass was, however, not associated with higher HF risk when compared with individuals with stable fat-free mass.

**Table 5:** Independent associations of changes in body composition measures and incident HF from baseline to imaging visit (mean 10 years from baseline visit) in analyses restricted to subset of participants with repeat body composition measures (57 670 participants) and without interval HF event before the imaging visit*

Body composition	⊿ % < –8% in body composition	−8% ≤ ⊿ % < –2% in body composition	Stable body composition –2% ≤ % change <2%	≥ 2% ⊿ %< 10% in body composition	≥ 10% ⊿ in body composition
	HR (95% CI)	HR (95% CI)	HR (95% CI)	HR (95% CI)	HR (95% CI)
Anthropometry					
Body mass index	1.35 (1.02 to 1.79)	1.07 (0.89 to 1.29)	1.00 (0.82 to 1.22)	0.87 (0.70 to 1.09)	1.96 (1.36 to 2.82)
Waist circumference	1.07 (0.81 to 1.41)	1.06 (0.871.29)	1.00 (0.79 to 1.27)	1.15 (0.96 to 1.39)	1.23 (0.89 to 1.71)
Waist-to-hip ratio	1.40 (0.96 to 2.05)	1.07 (.87 to 1.33)	1.00 (0.81 to 1.23)	1.19 (1.00 to 1.40)	1.04 (0.72 to 1.50)
Bioimpedance					
Body fat mass	1.10 (0.90 to 1.34)	1.33 (1.04 to 1.70)	1.00 (0.72 to 1.38)	0.96 (0.75 to 1.22)	0.89 (0.73 to 1.09)
Body fat-free mass	1.07 (0.77 to 1.50)	1.12 (0.96 to 1.30)	1.00 (0.82 to 1.23)	1.42 (1.08 to 1.87)	4.26 (2.12 to 8.55)

*HR estimates are floated absolute risks of HF and models were stratified by age at risk (in 5-year ranges), sex and UK region and adjusted for ethnicity, education, social deprivation, smoking, alcohol and physical activity.

HF, heart failure.

### Body fat distribution and HF LVEF subtypes

**Figure 2:**
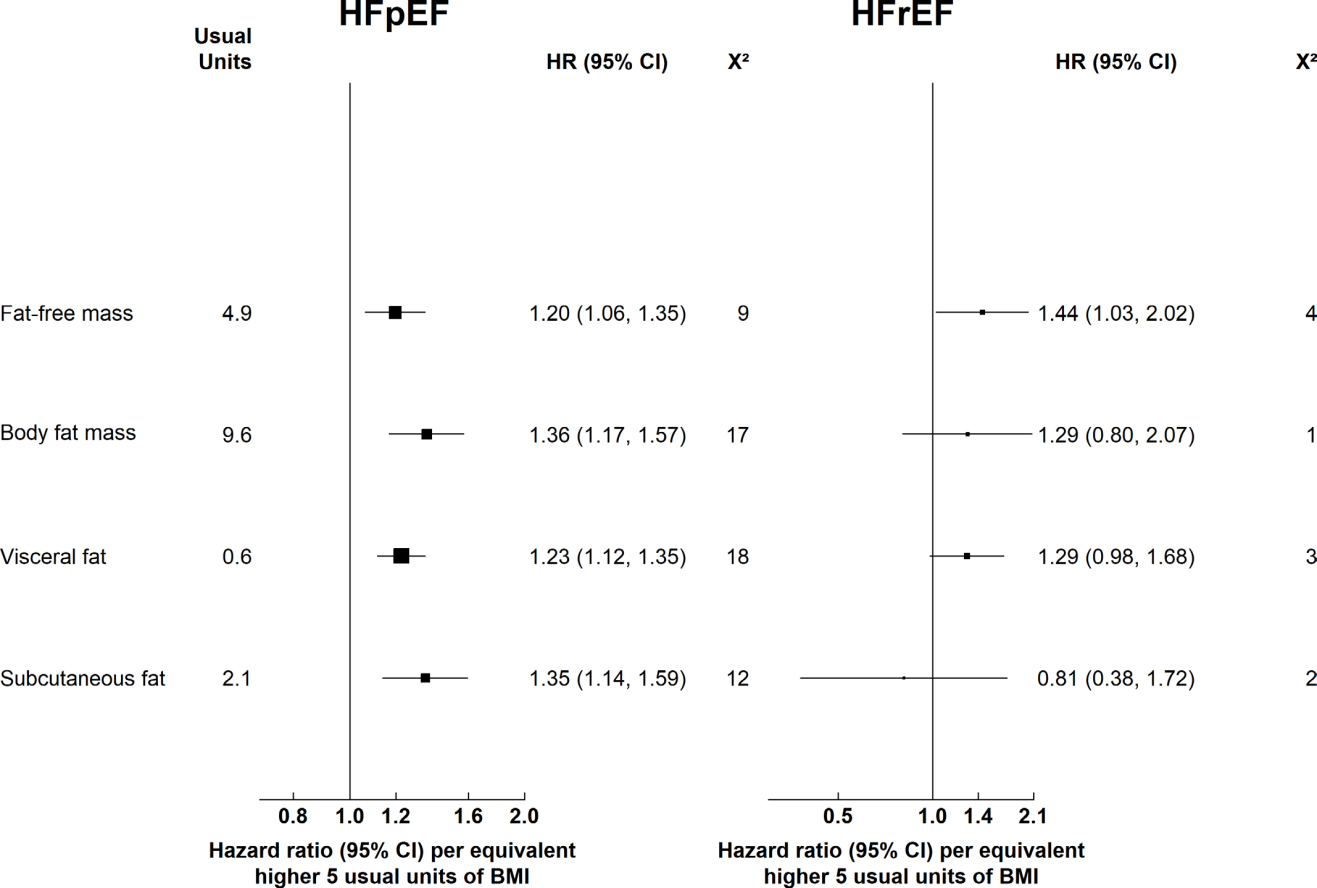
Associations of usual adiposity and fat measures with incident HF subtypes Cox regression models were stratified by age at risk (5-year age groups), sex and UK region and were adjusted for ethnicity, education, social deprivation, smoking, alcohol and physical activity. BMI, body mass index; HF, heart failure; HFpEF, HF with preserved ejection fraction; HFrEF, HF with preserved ejection fraction.

As shown in figure 2, DXA-derived fat-free mass was independently associated with both HFpEF (HR 1.20 (95% CI 1.06 to 1.35)) and HFrEF (HR 1.44 (95% CI 1.03 to 2.02)). Higher body fat mass was significantly associated with higher HFpEF risk (HR 1.36 (95% CI 1.17 to 1.57)) but was not associated with HFrEF risk (HR 1.29 (95% CI 0.80 to 2.07)). Excess VAT and SAT were significantly associated with HFpEF risk (23% and 35% higher risk respectively) but were not associated with HFrEF risk.

## Discussion

### Association shape and magnitude

In this large study, all body composition measures showed strong positive log-linear associations with incident HF across the range of distribution measured. The independent association with HF was stronger for central adiposity (WC) than general adiposity (BMI). Body fat mass was strongly associated with higher incident HF risk independent of fat-free mass while the association of fat-free mass with incident HF was largely explained by fat mass.

Previous studies have reported weaker associations for WC with respect to HF risk than described in the present report and tended to report stronger HF risk for general adiposity (BMI) than central adiposity (WC).^3^ The HUNT study^37^ and Cooper Centre Longitudinal study^38^ found a 27% and 28% higher HF risk, respectively, per 5 kg/m^2^ higher measured BMI. Similarly, some European cohorts such as the British Regional Heart Study,^39^ Rotterdam study,^40^ PREVEND study,^41^ Cohort of Swedish Men,^42^ Swedish Mammography Cohort^42^ and PPSWG study^43^ have reported risk estimates ranging from 10 to 40% higher HF risk per 5 kg/m^2^ higher measured BMI. However, Aune *et al* 3 in a meta-analysis of 23 studies on BMI and 12 studies on WC reported a 41% higher risk per 5 kg/m^2^ higher BMI and 29% higher risk per 10 cm higher WC.

Some other recent epidemiological studies have, however, shown that central adiposity measures like WC are more strongly associated with cardiovascular disease risk than BMI.^5 42 44–50^ The disparities between the findings of the studies may be partly explained by methodological problems such as reverse causation, measurement errors, poor quality data or non-exclusion of participants with cardiovascular diseases at baseline. In addition, none of the studies to date have corrected their results for measurement error and regression dilution bias. Despite the methodological differences in the cohort design between this analysis of the UKB and reports from other studies, the reported risk estimates in this study are comparable to other well-designed cohorts in Europe and the USA.^51–54^

This present study has shown that central adiposity is a stronger risk factor for HF than general adiposity, after adjustment for each other, in this middle-aged adult population. This is in keeping with findings from a few other studies.^55 56^ Nicklas *et al* 55 in the Health ABC study of community-dwelling Americans aged 70–79 years found that WC remained significantly associated with HF after adjustment for BMI (HR 1.27; 95% CI 1.04 to 1.54 per SD) while BMI was not significantly associated with HF after adjustment for WC (HR 1.08; 95% CI 0.86 to 1.35). A similar finding was reported from the Cardiovascular Health Study (CHS) of elderly Americans.^56^ Conversely, this present UKB study found a significant independent association of BMI (though weaker than WC) when adjusted for WC unlike the aforementioned studies. This may be because this study has a larger number of HF events to detect this association. Moreover, the American studies recruited older individuals.

Increase in BMI often reflects a complex interrelationship in the distribution of both fat mass and fat-free mass. Body fat mass is the total amount of adipose tissue in the body while fat-free mass is a surrogate for lean mass and includes all the body components except adipose tissue (ie, skeletal muscle mass, bone mineral mass, total body water and other organs).^57 58^ Body fat mass measured either by bioimpedance or DXA showed comparable strength of associations of risk of incident HF (~50% higher risk) while for fat-free mass, there was a weaker association. There is a dearth of studies that have examined the association of either fat mass or fat-free mass with HF risk. In the Health ABC,^51^ total body fat measured by DXA was associated with 31% higher HF risk per 8.76 kg increase.^51^ In a pooled analysis of two cohorts of elderly individuals from the CHS and Health ABC study, Zhang *et al* 57 did not find a significant association between any of the total fat mass and fat-free mass measured by DXA and incident HF in confounder-adjusted models. All the above studies had fewer HF events than the UKB and none of them evaluated the role of fat mass and fat-free mass independent of one another. In UKB, adjustment of body fat mass for fat-free mass did not substantially attenuate the observed association between body fat and incident HF, thus confirming that fat distribution independent of fat-free mass is more strongly associated with incident HF. To the best of our knowledge, no study has reported the independent associations of fat-free mass with incident HF. In the present study, fat-free mass showed weaker independent association with HF independent of fat mass. It could be argued that increased skeletal muscle mass and accompanying increase in blood volume could lead to higher BP and increased cardiac workload with consequent LV remodelling that predisposes to HF.^59^ However, higher fat-free mass also reflects higher visceral organs’ mass, non-fat soft tissue, fibrous tissue, bone mineral mass and extracellular water. Increased extracellular water and subclinical oedema could potentially explain the remaining excess risk of HF associated with higher fat-free mass. Indeed, in population studies, there is an inverse association between skeletal muscle mass and cardiovascular outcomes while increased extracellular fluid is associated with higher risk of cardiovascular outcomes.^60–65^ There is a need for studies in other populations to confirm the role of fat-free mass and its components, independent of body fat distribution in HF.

Few studies have investigated the independent association of VAT and SAT with incident HF. In the present study, both VAT and SAT were independently associated with higher risk of HF (~20% higher risk). This is consistent with recent findings by Rao *et al* 66 in the Jackson Heart Study (JHS). However, the Multi-ethnic Study of Atherosclerosis (MESA) study^16^ which had far fewer HF events than the UKB and JHS did not find a significant association for SAT despite the strong association seen for VAT with incident HF. Previous studies have found significant associations between each of VAT and SAT with cardiometabolic profile and LV function although stronger for VAT, suggesting that central fat distribution and subcutaneous fat in android and gynoid regions have metabolic activity that drives insulin resistance, inflammation and lipotoxicity that have been implicated in cardiac dysfunction.^67–80^ In our analyses of the joint association of general and central adiposity with incident HF, we have shown the log-linear increase in HF risk across tertiles of VAT, WC and WHR in normal weight, overweight and obese individuals. Although, HF risk increased log-linearly across categories of BMI regardless of central adiposity, the largest risk was seen in the top tertile of central fat distribution. Xu *et al* 81 in a recent analysis of the Atherosclerosis Risk in Communities (ARIC) study have also reported greater risk of HF with increasing tertiles of visceral adiposity index (calculated from WC and BMI). These findings corroborate the relevance of central fat distribution in cardiovascular risk. We have also recently shown in the UKB that VAT is the only body composition measure independently associated with LVEF after accounting for other body fat measures and fat-free mass.^82^

The stronger independent association of central adiposity with HF could be explained by the lipotoxic and depressive effects of VAT on myocardial fibres.^83^ Lipid accumulation also occurs in cardiomyocytes and epicardial tissue and leads to mitochondrial dysfunction and apoptosis of myocardial cells.^84 85^ This lipotoxicity has been associated with LV remodelling in the transition to HF.^83 86 87^

### Intermediate factors and causal mediators

In this study, cardiometabolic factors explained up to three-quarters of the associations between the body composition measures and incident HF. This is concurrent with previous studies that have investigated the role of cardiometabolic factors in cardiovascular disease risk.^88–90^ Increased adiposity is associated with elevated SBP, insulin resistance and a proatherogenic state that is associated with vascular inflammation and arterial stiffness.^91^ In turn, these factors may lead to LV remodelling and hypertrophy, followed by diastolic and systolic dysfunction, resulting in HF.^72 92–96^ This is especially true of central adiposity which has also been associated with increased sympathetic activity with attendant predisposition to tachyarrhythmias and HF.^97–99^

### Decadal changes in body composition measures and incident HF risk

We found a significantly increased HF risk for individuals with ≥10% decadal increase in BMI (2-fold higher risk) and individuals with ≥8% decadal decrease in BMI from baseline (35% higher HF risk) compared with individuals with stable BMI while decadal increase in body fat was also associated with higher HF risk. There was also significant graded increase in HF risk with decadal increase in fat-free mass. Decadal changes in WC and WHR were not significantly associated with incident HF risk. Few studies have assessed the associations between longitudinal changes in body composition measures and cardiovascular risk in the general population. Hu *et al* 34 in 1048 middle-aged individuals in Shanghai have reported a 2-fold higher risk of cardiovascular events in individuals with a 2-year ≥2% increase in body fat compared with individuals with stable body fat and 4-fold higher risk of cardiovascular events in those with more than 8% decrease in fat-free mass. Cheng *et al* 100 in an analysis of four US cohorts (MESA, ARIC, CHS and Framingham Heart Study) have reported a significant higher risk of HF with significant weight loss and weight gain which became noticeable 10 years before incident events. These findings point to the deleterious effects of both weight gain (both fat and fat-free mass) and weight loss for cardiovascular risk.

### Magnitude of associations in HF subtypes

The majority of studies on HF LV subtypes have come from cohorts in the USA^16 53 56 101 102^ and recently from the Dutch PREVEND cohort.^103^ Many of these studies have far fewer events than the UKB and the reported estimates are, therefore, more imprecise than UKB. We did not find a significant association between VAT or any of the other fat measures and HFrEF despite significant associations of these measures with HFpEF. Increased general and central adiposity has been associated with renin–angiotensin–aldosterone–system activation, resulting in greater blood volume, higher BP and elevated filling pressures, which causes increased LV mass and concentric hypertrophy, the hallmark of HFpEF.^91 104 105^ The stronger independent association of VAT with HFpEF could be explained by the pro-inflammatory and metabolic activity of VAT.^83^ Visceral adipose tissue, epicardial fat and vascular tissue secrete proinflammatory cytokines for example, TNFα, IL-1 and IL-6 which contribute to microvascular endothelial dysfunction and reduced vascular compliance.^69 70^ Reduced vascular compliance and a rise in intracardiac pressures exacerbates LV remodelling and hypertrophy leading to eventual myocardial burn-out.^106 107^ Systemic inflammation is upregulated in HF and detrimental to patients with HF and while inflammatory biomarkers are elevated in patients with HFpEF, this has not been well described in those with HFrEF, suggesting that this may not be a prominent pathway in HFrEF.^108^

### Future perspectives

The present study was not adequately powered to investigate the ethnic differences in the associations of body composition measures with incident HF. The UKB is a 95% European Caucasian cohort and participants of African and South-Asian ethnicities were not well represented in the cohort. Afro-Caribbeans have been shown to be at greater risk of HF than other ethnicities in population studies, however, this has often been attributed to the higher prevalence of hypertension in them.^109^ On the other hand, South-Asians have higher risk of coronary heart disease (CHD) and have more central adiposity and T2DM than other ethnicities in the UK.^110–114^ CHD is the most common aetiological subtype of HF in the UK and given the close link between adiposity and CHD in population studies, one would expect higher risk of adiposity-related HF events in people of South-Asian descent. Whether this is indeed the case in individuals of South-Asian descent remains unclear. The contributions of excess adiposity to HF risk in both ethnic minorities require further investigation.

The findings from the current study have important implications for population health. By demonstrating an independent relevance of fat distribution in addition to BMI on HF risk, our findings suggest that body fat imaging could be used for HF risk prediction in the general population. In addition, the added roles of different ectopic fat depots (epicardial fat, pericardial fat, intrahepatic fat, pancreatic fat and perinephric fat) in HF risk prediction in contemporary European populations and other ethnic groups require further research. Findings from such studies could contribute to HF risk prediction models by integrating fat imaging into current HF risk scores. Despite several population studies describing independent effects of central adiposity on HF outcomes beyond the BMI, many HF risk prediction scores do not currently include central adiposity measures and fat imaging.^5 6 103 115–125^ Risk prediction models for individuals without CVD in the general population are important as such models could aid the early identification of those at high risk of HF who would benefit from intensive primary and secondary prevention interventions (eg, weight reduction, physical activity, BP control).

### Strengths and limitations

To the best of our knowledge, this is the largest contemporary prospective cohort study with long follow-up that has investigated the association between multiple body composition measures and incident HF. We have been able to show the associations between body fat distribution and incident HF using different methods including direct fat imaging by DXA. Our findings provide insight into the potential role of fat and fat-free mass measurement in HF risk prediction in contemporary clinical practice. We have also been able to show the real-world associations of decadal changes in body composition measures and incident HF in a contemporary population free of cardiovascular disease. All measurements were done according to standard and validated methods. Correction for regression dilution bias addressed measurement error in the associations of different body composition measures with HF risk, unlike most previous studies. The electronic linkage of participants’ data to their health records allowed accurate ascertainment of HF events in this contemporary population. Moreover, excluding participants with major cardiac and vascular conditions at baseline limited the impact of reverse causation and confounding; the results were also robust to several sensitivity analyses.

The study is not without limitations. First, the number of participants in the underweight BMI category was modest (0.5%) and the study may not have been sufficiently powered to investigate the shape of the association for these participants. However, the proportion of underweight individuals in the study mirrors contemporary UK population and the absolute number of underweight participants in this analysis is substantially more than in other similar studies. The shape and strength of the observed association were similar for both fat mass and BMI and probably holds valid for these individuals. Second, BIA-derived fat and fat-free mass were estimated from proprietary algorithms which may not estimate regional fat and fat-free mass reliably, however, our analyses of DXA-derived measures were similar to the main analyses. Furthermore, while attempts have been made to reduce potential confounding, residual or unmeasured confounding is likely to remain. Fourth, there is limited ethnic diversity in the UKB (which is a predominantly white European cohort) and the findings from the study may not be generalisable to other ethnicities. Fifth, analyses of HF LV phenotypes were limited by the smaller number of individuals for which LV function was available in the cohort. Moreover, in Cox regression analyses of the risk of the HF subtypes, non-events would have included everyone else without LV data in the total sample and could have led to potential misclassification that cannot be quantified, as some people without available primary care data or LV imaging data could have had HF events which were not subtyped. Finally, it is not possible to take the reported observational associations as proof of causality but our findings corroborate recent evidence from Mendelian randomisation studies of the causality between BMI and incident HF.^126–129^

### Conclusion

In conclusion, adiposity, body fat distribution and fat-free mass are associated with a positive and log-linear risk of incident HF. Central fat distribution showed a stronger independent association for HF than the BMI. Body fat distribution was only independently associated with HFpEF and not HFrEF. These findings may be important for preventative population health strategies for tackling the current obesity pandemic.

## Data Availability

The data that support the findings of this study are available from the UK Biobank. The UK Biobank will make the data used for this study available to all bonafide researchers for health-related research that is in the public interest.
