# Supplementary material for: Adiposity, fat-free mass and incident heart failure in 500 000 individuals
Source: Open Heart. 2024 Jul 4;11(2):e002711. doi: 10.1136/openhrt-2024-002711 (PMC11227841; doi:10.1136/openhrt-2024-002711)
Supplement: Supplementary data [file openhrt-2024-002711supp001.pdf]

Table S1: Operational codes used to define incident heart failure

Disease category	Disease code category	Code definition
**ICD-10 HES and national death records**		
Hypertensive heart disease	I11	I11.0 Hypertensive heart disease with (congestive) heart failure
Hypertensive heart and renal disease	I13	I13.0 Hypertensive heart and renal disease with (congestive) heart failure<br>I13.2 Hypertensive heart and renal disease with both (congestive) heart failure and renal failure
Heart failure	I50	I50.0 Congestive heart failure<br>I50.1 Left ventricular failure<br>I50.9 Heart failure, unspecified
**OPCS 4**		
Transplantation of heart and lung	K01	K01 Transplantation of heart and lung<br>K01.1 Allotransplantation of heart and lung<br>K01.2 Revision of transplantation of heart and lung<br>K01.8 Other specified transplantation of heart and lung<br>K01.9 Unspecified transplantation of heart and lung
Other transplantation of heart	K02	K02 Other transplantation of heart<br>K02.1 Allotransplantation of heart NEC<br>K02.2 Xenotransplantation of heart<br>K02.3 Implantation of prosthetic heart<br>K02.4 Piggyback transplantation of heart<br>K02.5 Revision of implantation of prosthetic heart<br>K02.6 Revision of transplantation of heart NEC<br>K02.8 Other specified other transplantation of heart<br>K02.9 Unspecified other transplantation of heart
Open heart assist operations	K54	K54 Open heart assist operations<br>K54.1 Open implantation of ventricular assist device<br>K54.2 Open removal of ventricular assist device<br>K54.8 Other specified open heart assist operations<br>K54.9 Unspecified open heart assist operations
Transluminal heart assist operations	K56	K56 Transluminal heart assist operations<br>K56.1 Transluminal insertion of pulsation balloon into aorta<br>K56.2 Transluminal insertion of heart assist system NEC<br>K56.3 Transluminal maintenance of heart assist system<br>K56.4 Transluminal removal of heart assist system<br>K56.8 Other specified transluminal heart assist operations<br>K56.9 Unspecified transluminal heart assist operations
Transluminal operations on cardiac conduit	K76	K76 Transluminal operations on cardiac conduit<br>K76.1 Percutaneous transluminal balloon dilation of cardiac conduit<br>K76.8 Other specified transluminal operations on cardiac conduit<br>K76.9 Unspecified transluminal operations on cardiac conduit

HES: Hospital Episodes Statistics, **OPCS:** Office of Population Censuses and Surveys.

Table S2: Diagnosis codes for HF ejection fraction subtypes in primary care

Heart failure subtype	Code type	Diagnosis codes
HFpEF	Read codes	585g<br>G5yyA<br>G5yyC<br>G583.<br>XaJ99<br>XaItG<br>XaYYs<br>XaWyi<br>585k.<br>XaJvY<br>585R.<br>58530<br>5C20.<br>XaJKz<br>XaItG Left ventricular diastolic dysfunction<br>XaJ99 Echocardiogram shows left ventricular diastolic dysfunction<br>XaWyi Heart failure with normal ejection fraction<br>XaYYs Diastolic dysfunction
	SNOMED CT	"3545003"<br>"418304008"<br>"443343001"<br>"441530006"<br>"120891000119109"<br>"120881000119106"<br>"443344007"<br>"446221000"<br>"395704004"<br>"441530006"<br>"443344007"
HFrEF	Read codes	585f.<br>G5yy9<br>XaJ98<br>XaIIq<br>XafeB<br>G5yyD<br>G581.13<br>33BA.<br>XM1Qn Impaired left ventricular function<br>XaIIq Left ventricular systolic dysfunction<br>XaJ98 Echocardiogram shows left ventricular systolic dysfunction<br>XafeB Heart failure with reduced ejection fraction
	SNOMED CT	"441481004"<br>"153931000119109"<br>"153951000119103"<br>"153941000119100"<br>"442304009"<br>"134401001"<br>"698592004"<br>"430396006"<br>"443253003"<br>"371037005"<br>"417996009"<br>"443254009"<br>"120861000119102"<br>"120851000119104"<br>"15629741000119102"<br>"15629641000119107"<br>"703272007"<br>"703275009"<br>"703273002<br>"703276005"<br>"703274008"

Table S3: Age-adjusted sex-specific partial correlation coefficients between body composition measures at baseline visit*†

		WC	WHR	Body fat mass	Body fat-free mass
**BMI**	**Women**	0.87	0.45	0.94	0.69
	**Men**	0.88	0.60	0.92	0.65
**WC**		**Women**	0.74	0.88	0.66
		**Men**	0.80	0.89	0.65
**WHR**			**Women**	0.44	0.28
			**Men**	0.61	0.32
**Body fat mass**				**Women**	0.71
				**Men**	0.60

*Analysis among 428,087 UKB participants included in the main analysis.
†Pearson partial correlation coefficients are reported in this table. BMI: body mass index; WC: waist circumference; WHR: waist hip ratio.

Oguntade AS, *et al. Open Heart* 2024; 11:e002711. doi: 10.1136/openhrt-2024-002711

Table S4: Age-adjusted sex-specific partial correlation coefficients between DXA-derived fat measures at baseline imaging visit[*†]

		Visceral fat	Subcutaneous fat	Fat-free mass
**Body fat**	**Women**	0.82	0.96	0.53
	**Men**	0.87	0.93	0.44
**Visceral fat**		**Women**	0.67	0.40
		**Men**	0.65	0.37
**Subcutaneous fat**			**Women**	0.52
			**Men**	0.41

[*]Analysis among 36,278 UKB participants (men=17309; women=18969) included in the DXA-imaging analysis. [†]Pearson partial correlation coefficients are reported in this table. BMI: body mass index; WC: waist circumference; WHR: waist hip ratio.

Oguntade AS, *et al. Open Heart* 2024; 11:e002711. doi: 10.1136/openhrt-2024-002711

Table S5: Regression Dilution Ratios (RDR) of body composition measures

Body composition	Women*	Men*	All†
**Anthropometric and bio-impedance measures**			
BMI	0.92	0.93	0.92
Waist circumference	0.82	0.82	0.82
Waist-hip ratio	0.65	0.66	0.65
Body fat mass	0.90	0.90	0.90
Body fat-free mass	0.91	0.95	0.94
**DXA**			
Visceral fat mass	0.93	0.92	0.92
Subcutaneous fat mass	0.93	0.92	0.93
Body fat mass	0.93	0.93	0.93
Body fat-free mass	0.97	0.98	0.97

Number of resurvey participants=17,450 (men=8286; women-=9164) for anthropometric and bio-impedance measures while number of resurvey participants=2913 (men=1425; women-=1488) for DXA measures. *adjusted for 5year age-group; †adjusted for 5 year age-group and sex.

Table S6: Baseline characteristics of UK Biobank participants by sex*

Characteristics at baseline<br>Number of participants	Women<br>N=239,775	Men<br>N=188,312	Total<br>N=428,087
**Demographic and lifestyle factors**			
Age (years)	55.9 (8.0)	55.9 (8.2)	55.9 (8.1)
Recruitment regions			
England	212,710 (88.7%)	167,525 (89.0%)	380,235 (88.8%)
Wales	9,870 (4.1%)	7,843 (4.2%)	17,713 (4.1%)
Scotland	17,195 (7.2%)	12,944 (6.9%)	30,139 (7.0%)
Ethnicity			
White	227,100 (94.7%)	178,077 (94.6%)	405,177 (94.6%)
Asian	3,970 (1.7%)	4,147 (2.2%)	8,117 (1.9%)
African	3,898 (1.6%)	2,946 (1.6%)	6,844 (1.6%)
others	4,807 (2.0%)	3,142 (1.7%)	7,949 (1.9%)
Higher education	153,256 (63.9%)	123,606 (65.6%)	276,862 (64.7%)
Most deprived	47,030 (19.6%)	38,480 (20.4%)	85,510 (20.0%)
Current smoker	20,911 (8.7%)	23,108 (12.3%)	44,019 (10.3%)
Regular/daily drinker	152,909 (63.8%)	147,873 (78.5%)	300,782 (70.3%)
Low physical activity	42,807 (17.9%)	31,809 (16.9%)	74,616 (17.4%)
**Anthropmetry**			
BMI (kg/m^2)	26.9 (5.0)	27.7 (4.1)	27.2 (4.7)
Waist circumference (cm)	84.2 (12.2)	96.4 (10.9)	89.6 (13.1)
Hip circumference (cm)	103.1 (10.1)	103.2 (7.3)	103.1 (9.0)
Waist-hip ratio	0.81 (0.07)	0.93 (0.06)	0.87 (0.09)
Waist-height ratio	0.52 (0.08)	0.55 (0.06)	0.53 (0.07)
**Bioimpedance**			
Body fat percentage (%)	36.4 (6.8)	25.0 (5.7)	31.4 (8.5)
Whole body fat mass (kg)	26.6 (9.8)	21.9 (8.0)	24.5 (9.4)
Whole body fat-free mass (kg)	44.5 (4.9)	63.7 (7.7)	52.9 (11.4)
Trunk fat percentage (%)	33.9 (7.7)	27.3 (6.5)	31.0 (7.9)
Trunk fat mass (kg)	13.5 (5.2)	13.6 (4.8)	13.5 (5.0)
Trunk fat-free mass (kg)	25.0 (2.6)	35.1 (3.9)	29.4 (5.9)
**†DXA imaging visit**			
DXA body fat mass	26.4 (9.6)	24.6 (8.8)	25.5 (9.3)
DXA visceral fat	0.8 (0.6)	1.7 (1.0)	1.2 (0.9)
DXA-subcutaneous adipose tissue (kg)	6.0 (2.1)	4.5 (1.6)	5.3 (2.0)
DXA fat-free mass (kg)	41.9 (4.9)	58.4 (6.7)	49.8 (10.1)
**Medical conditions**			
Hypertension	52,564 (21.9%)	51,603 (27.4%)	104,167 (24.3%)
Diabetes	7,869 (3.3%)	10,637 (5.6%)	18,506 (4.3%)
Chronic kidney disease	1,328 (0.6%)	1,131 (0.6%)	2,459 (0.6%)
BP lowering medication usage	35,916 (15.0%)	35,136 (18.7%)	71,052 (16.6%)
Insulin usage	1,522 (0.6%)	2,009 (1.1%)	3,531 (0.8%)
Lipid lowering medication usage	22,968 (9.6%)	29,212 (15.5%)	52,180 (12.2%)
Systolic blood pressure (mmHg)	135.0 (19.1)	141.1 (17.3)	137.7 (18.6)
Diastolic blood pressure (mmHg)	80.8 (9.9)	84.6 (9.9)	82.4 (10.1)
**Blood biomarkers**			
Glucose (mmol/L)	5.0 (1.0)	5.1 (1.3)	5.1 (1.2)
HbA1c (mmol/mol)	35.6 (5.6)	36.0 (7.0)	35.8 (6.3)
HDL-C (mmol/L)	1.6 (0.4)	1.3 (0.3)	1.5 (0.4)
LDL-C (mmol/L)	3.7 (0.9)	3.6 (0.8)	3.6 (0.8)
eGFR (ml/min)	96.8 (16.3)	84.2 (14.7)	91.2 (16.8)
‡C-reactive protein (mg/L)	2.8 (0.7)	2.9 (0.6)	2.8 (0.7)

* Data are presented as arithmetic mean (SD), geometric mean (SD) or n(%);
‡Geometric mean (SD); BMI: body mass index; eGFR: estimated glomerular filtration rate; †Imaging visit occurred 10 years after the baseline visit.

Oguntade AS, et al. Open Heart 2024; 11:e002711. doi: 10.1136/openhrt-2024-002711

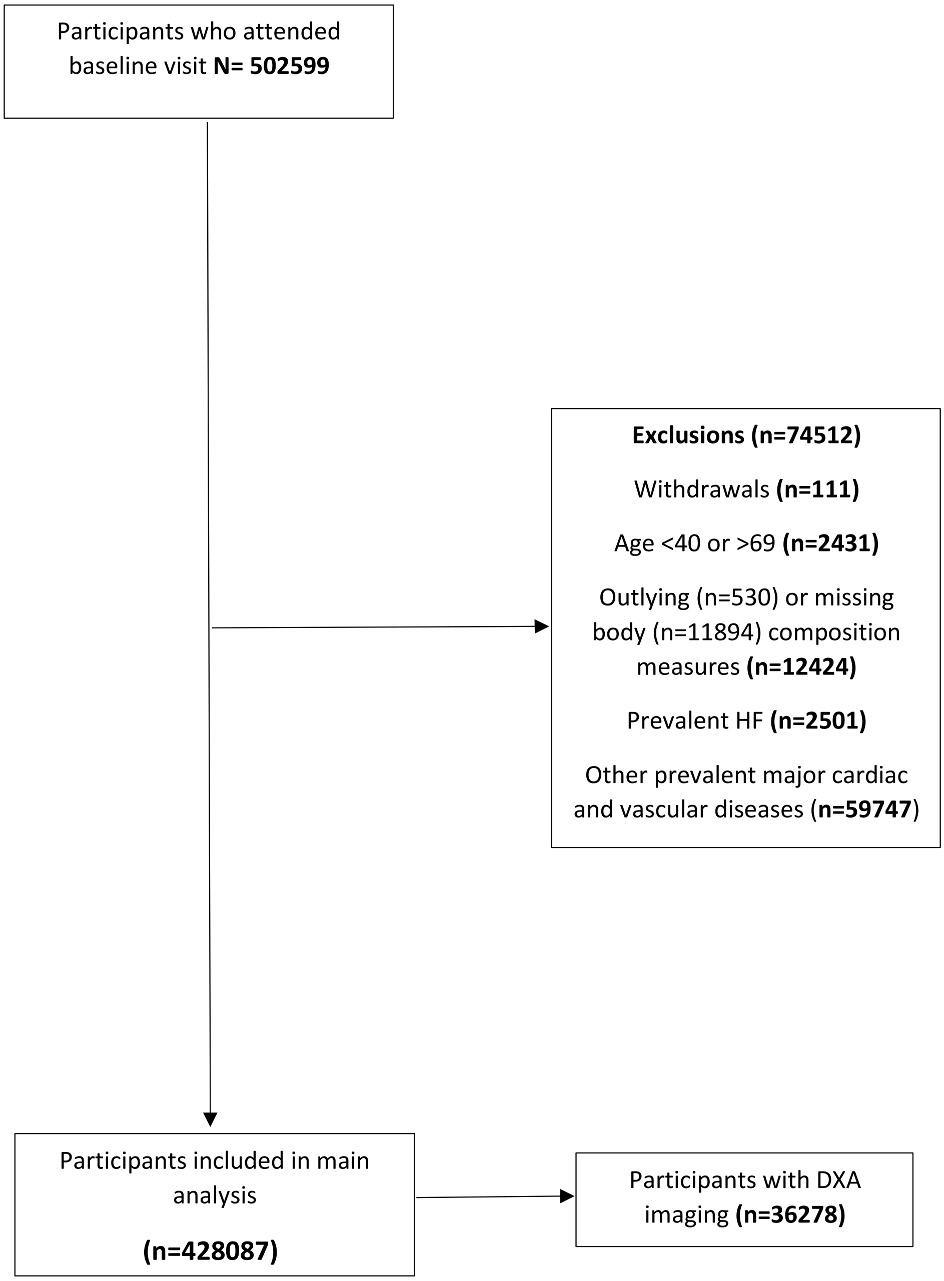

Figure S1: Flow diagram of UK Biobank study population

Oguntade AS, *et al. Open Heart* 2024; 11:e002711. doi: 10.1136/openhrt-2024-002711

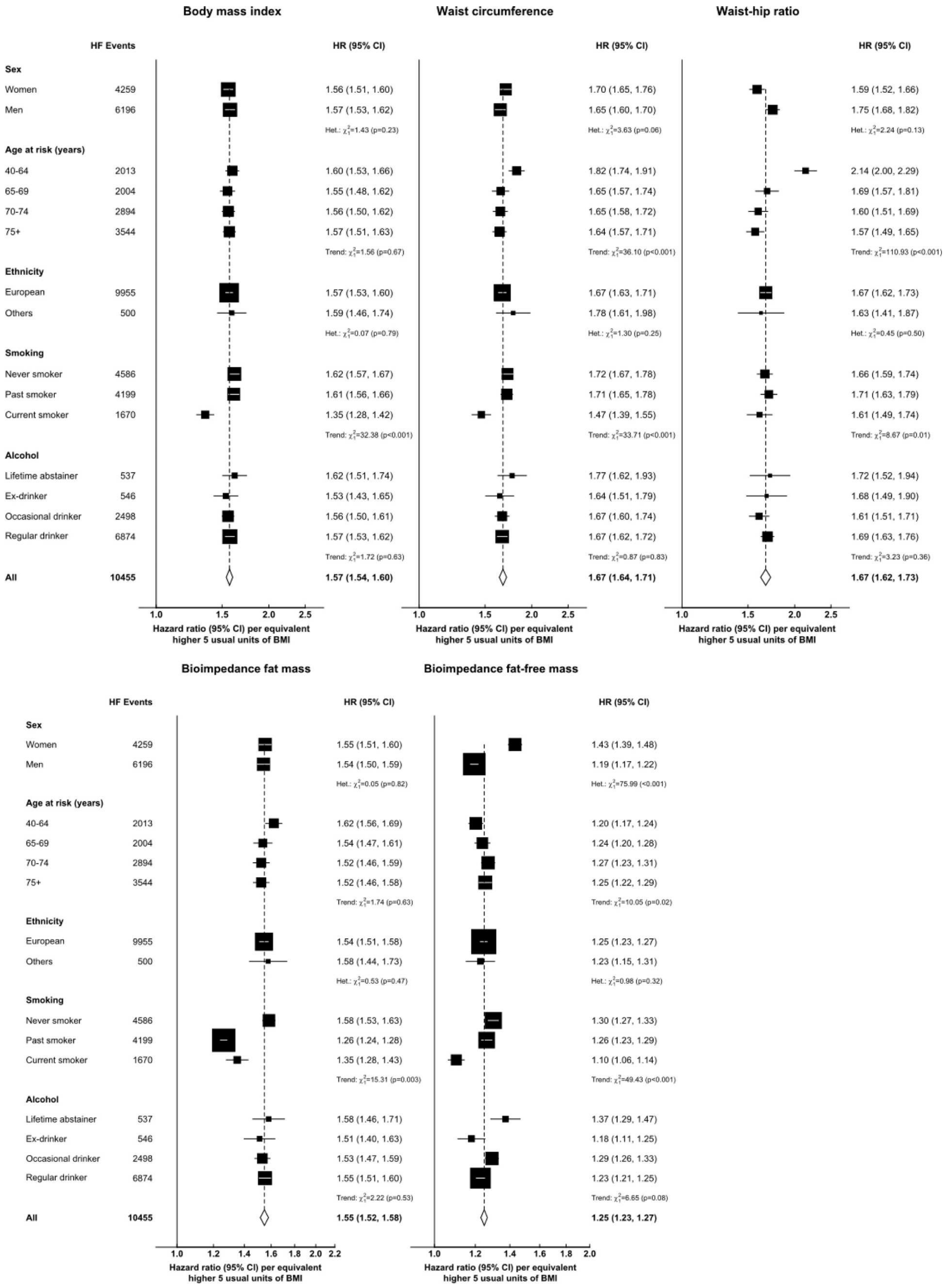

Figure S2: Hazard ratios (95% CI) of HF per 5 usual BMI units equivalent of anthropometric and bio-impedance measures by potential confounders

Box area is inversely proportional to the variance of the log risk. Where appropriate, hazard ratio (HR) estimates are stratified by age at risk (in 5-year ranges), sex and UK region, and are adjusted for ethnicity, education, social deprivation, smoking, alcohol and physical activity.

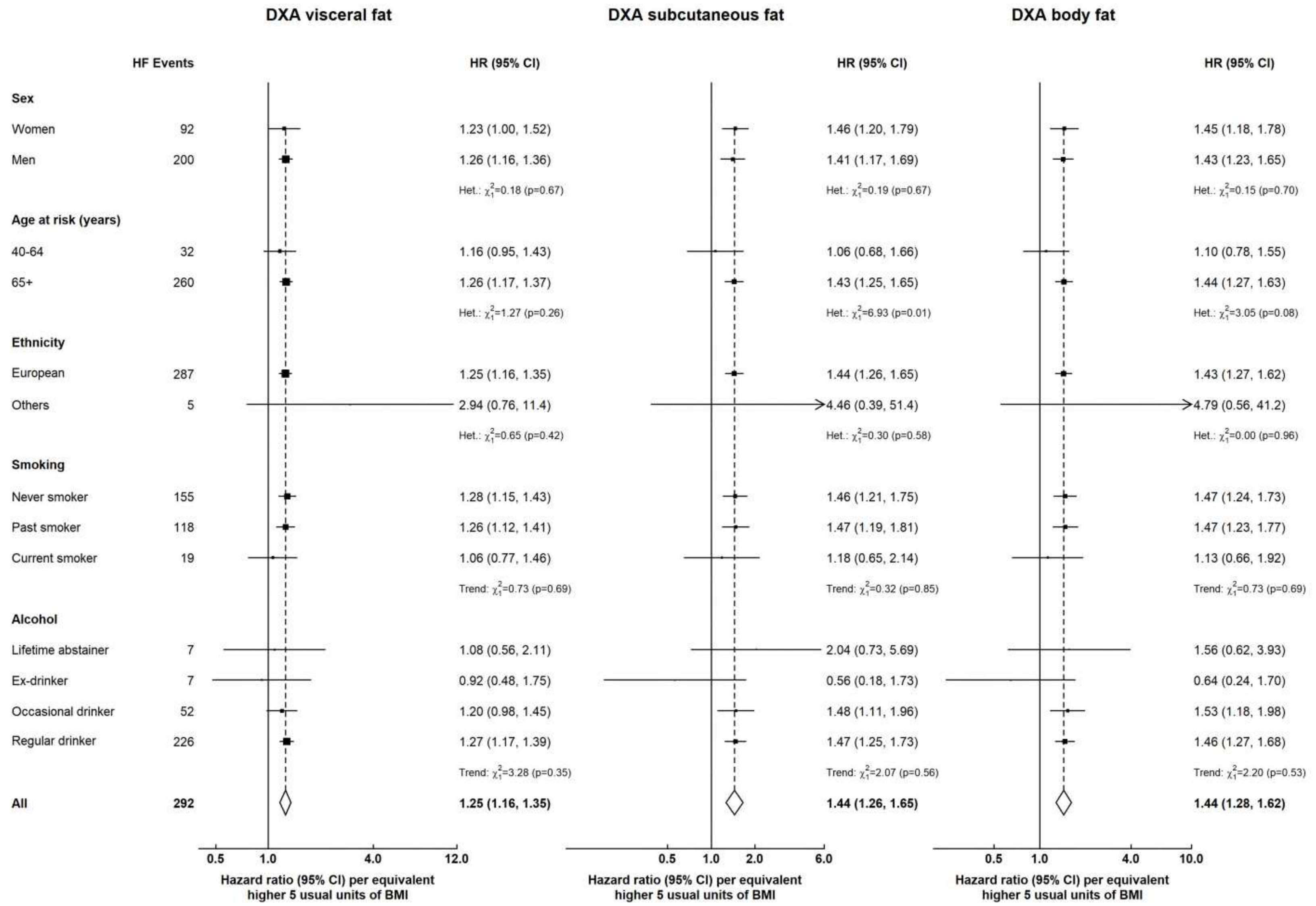

Figure S3: Hazard ratios (95% CI) of HF per 5 usual BMI units equivalent of DXA fat measures by potential confounders

Box area is inversely proportional to the variance of the log risk. Where appropriate, hazard ratio (HR) estimates are stratified by age at risk (in 5-year ranges), sex and UK region, and are adjusted for ethnicity, education, social deprivation, smoking, alcohol and physical activity.

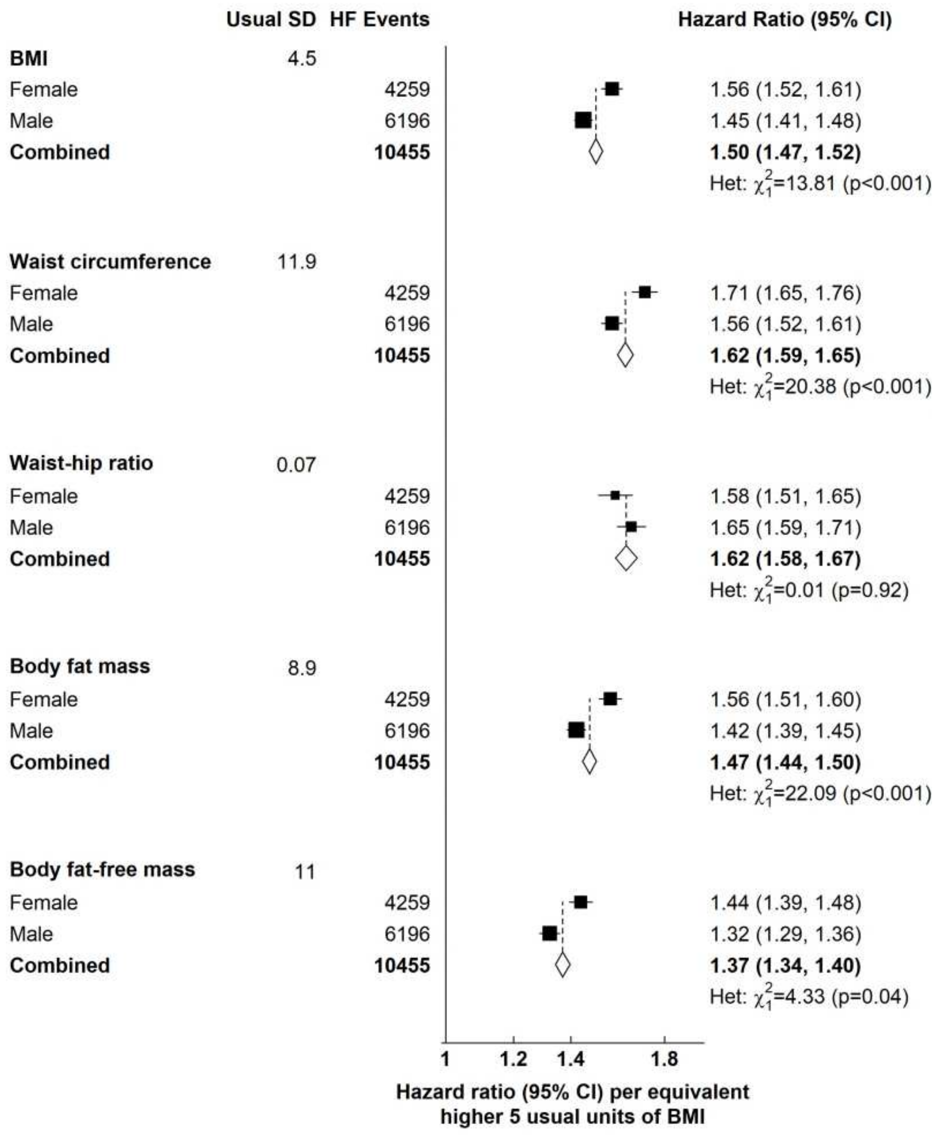

Figure S4: Hazard ratios (95% CI) of HF per usual SD of anthropometric and bio-impedance measures in main analyses

Box area is inversely proportional to the variance of the log risk. Where appropriate, hazard ratio (HR) estimates are stratified by age at risk (in 5-year ranges), sex and UK region, and are adjusted for ethnicity, education, social deprivation, smoking, alcohol and physical activity.

Oguntade AS, *et al. Open Heart* 2024; 11:e002711. doi: 10.1136/openhrt-2024-002711

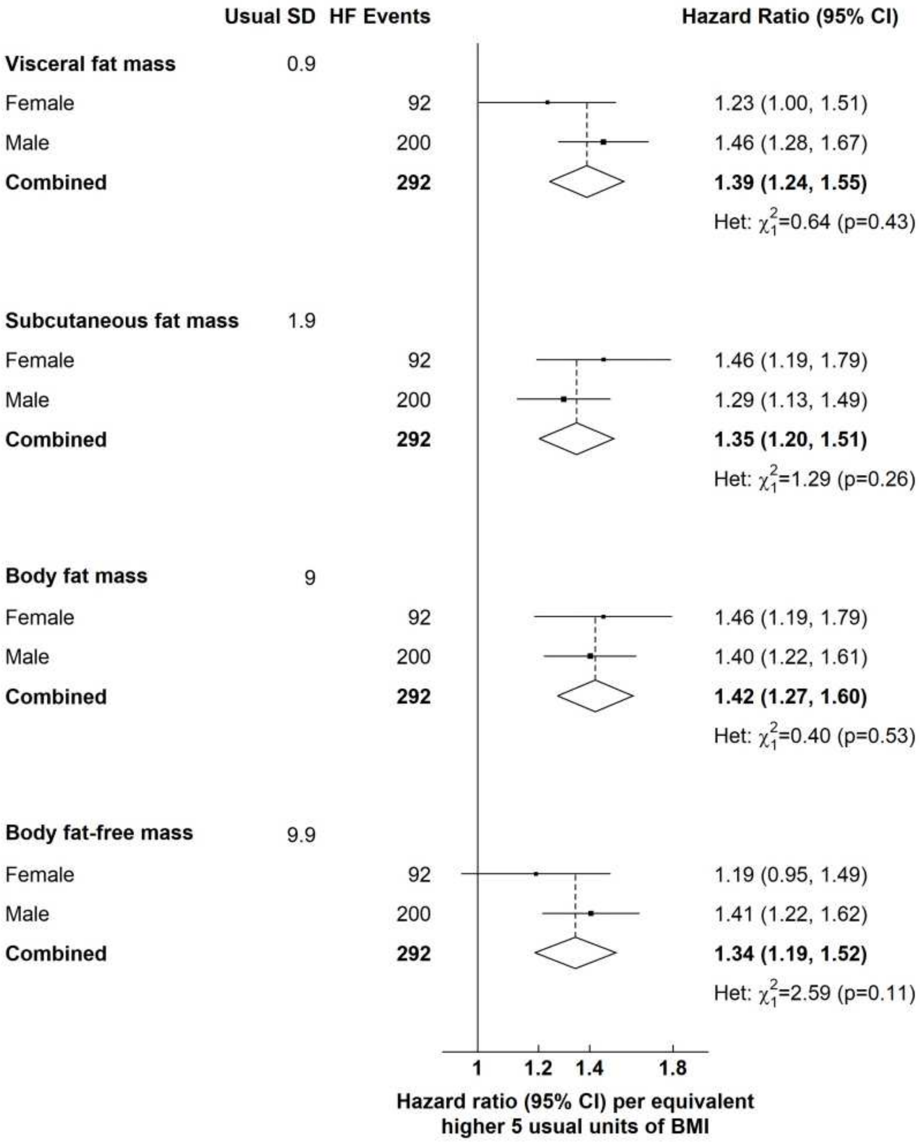

Figure S5: Hazard ratios (95% CI) of HF per usual SD of DXA-derived measures in supplementary analyses

Box area is inversely proportional to the variance of the log risk. Where appropriate, hazard ratio (HR) estimates are stratified by age at risk (in 5-year ranges), sex and UK region, and are adjusted for ethnicity, education, social deprivation, smoking, alcohol and physical activity.

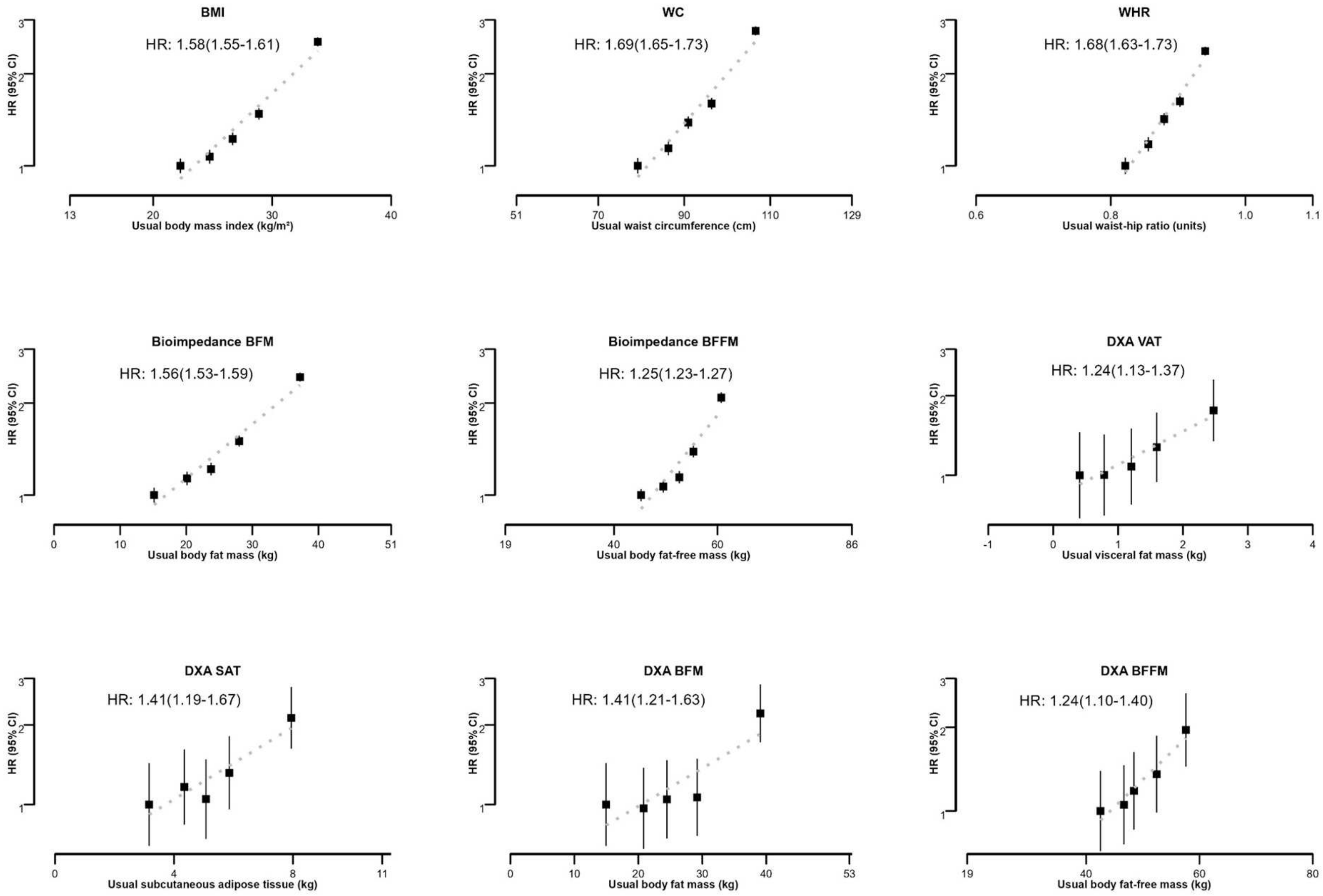

Figure S5: Hazard ratios (95% CI) of incident HF with increasing usual units of body composition measures in all participants excluding first 2 years of follow-up.

Error bars denote group-specific 95% CIs. Hazard ratio estimates are stratified by age at risk (in 5-year ranges), sex and UK region, and are adjusted for ethnicity, education, social deprivation, smoking, alcohol and physical activity

Oguntade AS, *et al. Open Heart* 2024; 11:e002711. doi: 10.1136/openhrt-2024-002711

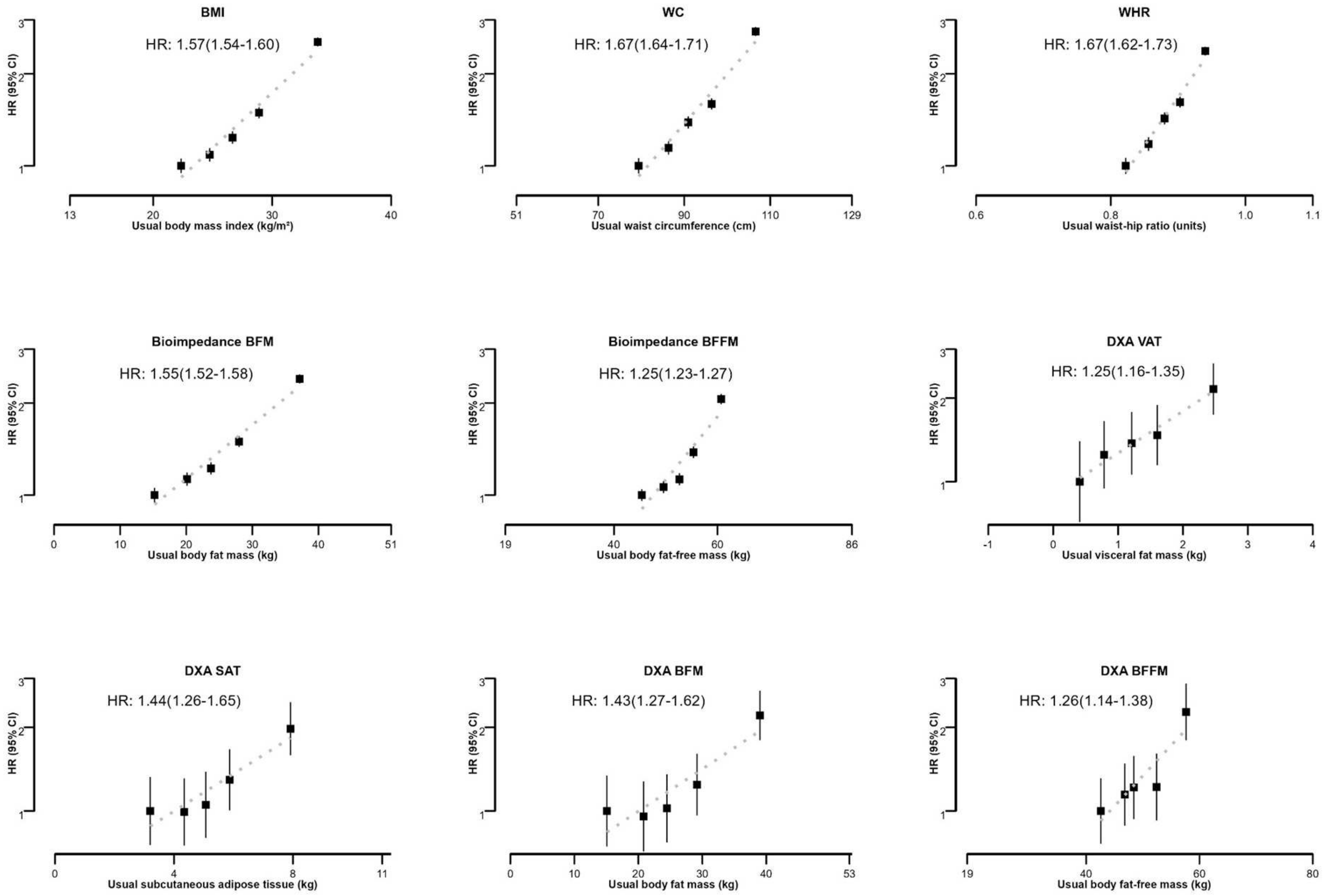

Figure S6: Hazard ratios (95% CI) of incident HF with increasing usual units of body composition measures in all participants excluding underweight participants

Error bars denote group-specific 95% CIs. Hazard ratio estimates are stratified by age at risk (in 5-year ranges), sex and UK region, and are adjusted for ethnicity, education, social deprivation, smoking, alcohol and physical activity

Oguntade AS, *et al. Open Heart* 2024; 11:e002711. doi: 10.1136/openhrt-2024-002711

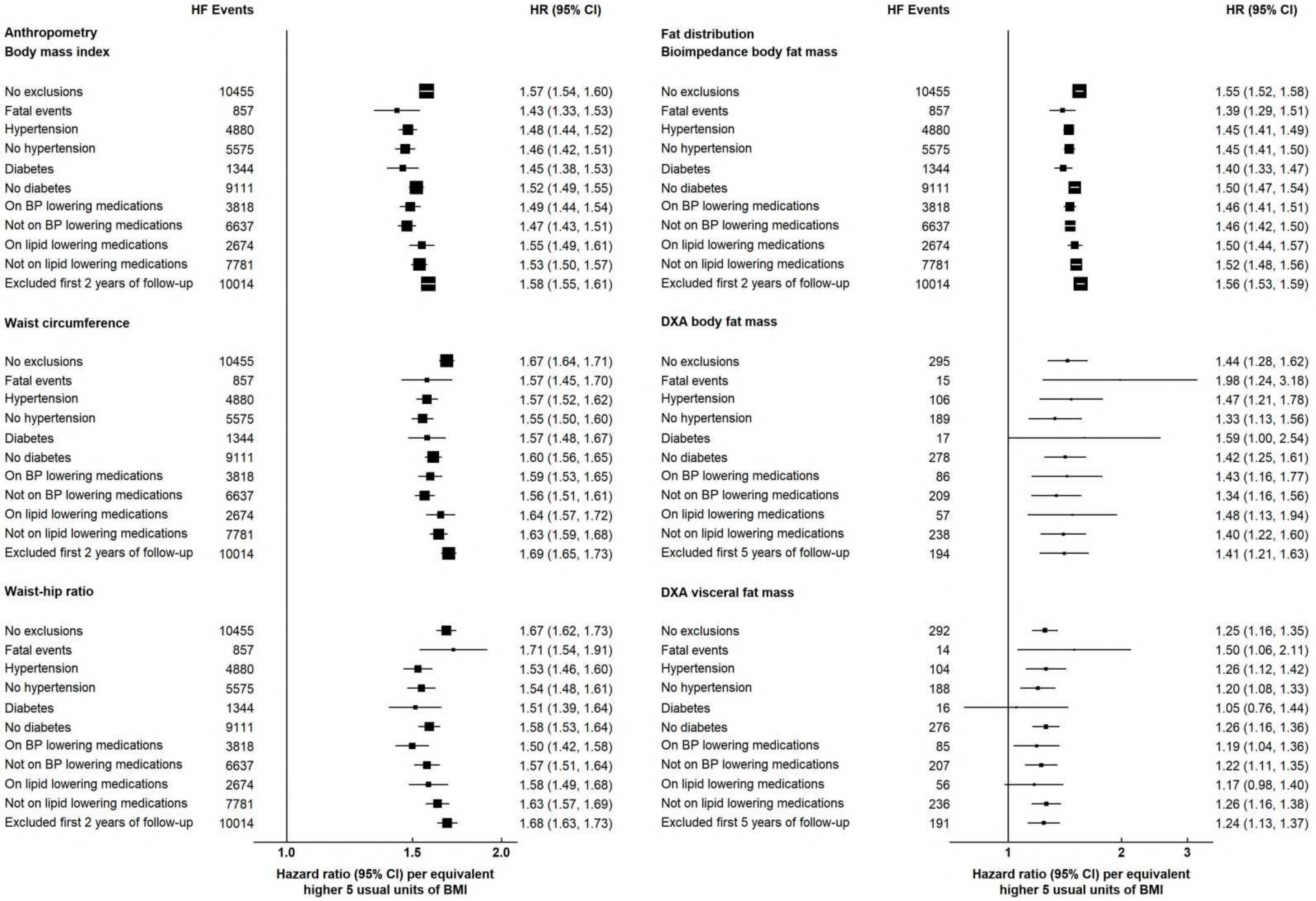

Figure S7: Hazard ratios of HF per equivalent 5 BMI usual units higher body composition measures before and after various exclusions

Box area is inversely proportional to the variance of the log risk. Where appropriate, hazard ratio (HR) estimates are stratified by age at risk (in 5-year ranges), sex and UK region, and are adjusted for ethnicity, education, social deprivation, smoking, alcohol and physical activity.

Oguntade AS, *et al. Open Heart* 2024; 11:e002711. doi: 10.1136/openhrt-2024-002711
